# Synergistic Charge Transfer Effect in Ferrous Heme–CO Bonding within Cytochrome P450

**DOI:** 10.3390/molecules29040873

**Published:** 2024-02-16

**Authors:** Enhua Zhang, Hajime Hirao

**Affiliations:** Warshel Institute for Computational Biology, School of Medicine, The Chinese University of Hong Kong, Shenzhen 518172, China; 223055007@link.cuhk.edu.cn

**Keywords:** cytochrome P450, carbon monoxide, coordination bonding, charge transfer, valence bond theory

## Abstract

We conducted ab initio valence bond (VB) calculations employing the valence bond self-consistent field (VBSCF) and breathing orbital valence bond (BOVB) methods to investigate the nature of the coordination bonding between ferrous heme and carbon monoxide (CO) within cytochrome P450. These calculations revealed the significant influence exerted by both proximal and equatorial ligands on the π-backdonation effect from the heme to the CO. Moreover, our VB calculations unveiled a phenomenon of synergistic charge transfer (sCT). In the case of ferrous heme–CO bonding, the significant stabilization in this sCT arises from cooperative resonance between the VB structures associated with σ donation and π backdonation. Unlike many other ligands, CO possesses the unique ability to establish two mutually perpendicular π-backdonation orbital interaction pairs, leading to an intensified stabilization attributed to σ–π resonance. Furthermore, while of a smaller energy magnitude, sCT due to one π–π pair is also present, contributing to the differential stabilization of ferrous heme–CO bonding.

## 1. Introduction

The interaction between the ferrous heme and carbon monoxide (CO) stands as one of the most fundamental types of coordination bonds found in cytochrome P450 enzymes (P450s). The distinctive spectral features displayed by the ferrous–CO complex of P450s not only served as the inspiration behind the unique nomenclature “P450” but also played a crucial role in the initial discovery and characterization of these enzymes [1,2,3]. The nature of ferrous heme–CO bonding in P450s and other heme proteins has been extensively studied using techniques such as infrared and resonance Raman spectroscopy [4,5]. Furthermore, the strong affinity of ferrous heme for CO remains a widely utilized phenomenon in contemporary experimental investigations, including CO binding assays and deliberate enzyme inhibition strategies [6,7,8].

**Figure 1:**
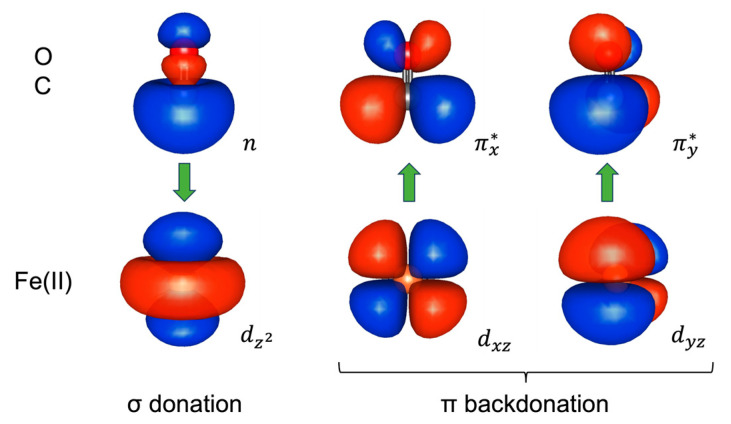
Schematic illustration of one σ-donation and two π-backdonation orbital-interaction pairs for CT in ferrous heme–CO bonding.

At the core of ferrous heme–CO bonding lies the pivotal role played by bidirectional charge transfer (CT) [4,5,9,10,11]. Among the array of ligands capable of binding to the P450 heme, CO distinguishes itself due to its ability to establish two mutually perpendicular pairs of orbital interactions for π-backdonation CT, as depicted in Figure 1. To quantitatively assess the effects of σ donation and π backdonation, as well as to determine their relative significance—unattainable through spectroscopic studies—we have recently conducted comprehensive theoretical studies employing complementary occupied–virtual orbital pair (COVP) and quantum theory of atoms-in-molecules (QTAIM) analyses based on density functional theory (DFT) [10,12,13]. Through these analyses, we have convincingly demonstrated that π backdonation holds greater significance than σ donation in the ferrous heme–CO complex within P450s. Furthermore, our more recent research has shown that the energetic stabilization for π backdonation in the ferrous heme–CO complex surpasses what could be expected from the amount of transferred electron density [11]. This intriguing observation suggests the presence of additional stabilization in the ferrous heme–CO complex when compared to other heme–ligand complexes. Thus, the nature of ferrous heme–CO bonding in P450s presents an intellectually captivating subject, worthy of renewed scrutiny.

In our quest to unravel the quantum mechanical foundations of ferrous heme–CO coordination bonding in P450s, this study leverages ab initio valence bond (VB) theory. Beyond its indisputable role in advancing our comprehension of chemistry after the establishment of quantum mechanics [14], VB theory has undergone significant development in recent decades, becoming a quantitatively competitive ab initio tool [15,16,17]. As it has evolved, ab initio VB theory has extended its application beyond organic molecules, providing invaluable insights into donor–acceptor and metal–ligand interactions [18,19,20,21,22,23,24,25]. Furthermore, VB calculations have brought to light a novel phenomenon termed charge-shift bonding, significantly enriching our comprehension of chemical bonds [26,27]. The driving force behind charge-shift bonding defies conventional covalent bonding models, with resonance between covalent and ionic VB structures emerging as a prominent factor facilitating bonding. The potential and utility of ab initio VB theory are poised for further expansion into the realm of complex biological systems containing transition metals, such as P450s.

This study pursues two primary objectives: firstly, to evaluate how proximal and equatorial ligands influence the relative significance of σ donation and π backdonation within the framework of bidirectional CT in P450s; and secondly, to elucidate the underlying mechanisms responsible for the observed additional CT stabilization in the ferrous heme–CO complex.

## 2. Results and Discussion

### 2.1. Wave Function Analysis: Relative Significance of Different VB Structures

**Scheme 1:**
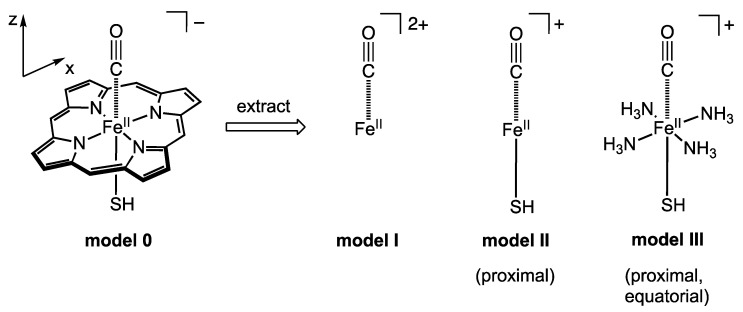
Models I–III for VB analysis, constructed utilizing the previously optimized geometry of a P450 model [10].

**Figure 2:**
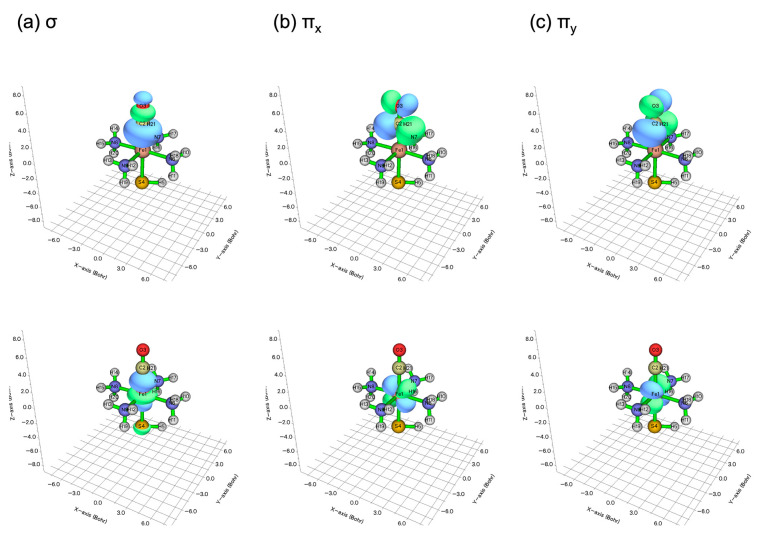
VB orbitals in the (**a**) σ, (**b**) π_x_, and (**c**) π_y_ frameworks, obtained through the VBSCF-VB(all) calculation for model III.

In our VB analysis of ferrous heme–CO bonding, we employed the three models (I–III) depicted in Scheme 1. Further details regarding the model setup can be found in the Materials and Methods section (Section 3). Figure 2 displays the hybrid atomic orbitals (AOs) associated with the σ-donation and π-backdonation bonding pairs in model III, as determined through the VB(all) calculation with the valence bond self-consistent field (VBSCF) method. Similar orbitals were obtained for models I and II (Figures S2 and S3). These orbitals align with the conventional understanding of σ donation occurring from the *n*(CO) orbital to the $d_{z^{2}}\left(\mathrm{Fe}\right)$ orbital and π backdonation from the $d_{xz}\left(\mathrm{Fe}\right)\;$and $d_{yz}\left(\mathrm{Fe}\right)$ orbitals to the π*(CO) orbitals (Figure 1).

**Scheme 2:**
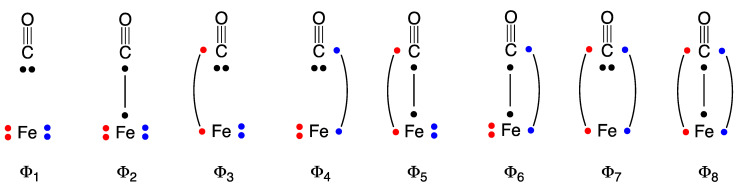
Schematic drawings of representative VB structures. Black, red, and blue colors are used for electrons involved in σ donation, π backdonation in the *xz* plane, and π backdonation in the *yz* plane, respectively.

Table 1 presents the weights (*W*_K_) of the representative VB structures illustrated in Scheme 2 (see also Section 3 for theoretical details). The weights were calculated from the VB structure coefficients and the overlap integrals between VB structures, using the Coulson–Chirgwin formula [28]. A noteworthy observation from the table is that VBSCF-calculated *W*_1_ consistently demonstrates the largest weight of Φ_1_ (68.39% in model I, 69.26% in model II, and 50.25% in model III) (Table 1a). In model I, *W*_2_ is the second largest and significantly larger than the *W*_3_ and *W*_4_ values. These results suggest that σ donation prevails over π backdonation in this model. In model II, *W*_2_ decreases while *W*_3_ and *W*_4_ increase compared to their corresponding weights in model I, indicating that the proximal HS^–^ ligand attenuates σ donation while enhancing π backdonation. The attenuation of σ donation and the enhancement of π backdonation are even more pronounced in model III, where *W*_2_–*W*_4_ exhibit similar values (8.59, 8.52, and 8.53%, respectively), underscoring the role of equatorial ligands in promoting π backdonation. Another intriguing finding for model III is the relatively large values of *W*_5_ and *W*_6_ (9.27%), indicating that states concurrently involving one Heitler–London (HL) bonding pair for σ donation and one for π backdonation emerge as significant contributors to the overall wave function. Breathing orbital valence bond (BOVB) calculations produced results qualitatively similar to those obtained from VBSCF calculations (Table 1b). However, the BOVB decreased the weights of Φ_1_ while increasing the weights of Φ_2_–Φ_4_, suggesting that the orbital breathing effect enhances CT in both directions. *W*_7_ and *W*_8_ were found to be very small in both the VBSCF and BOVB calculations.

To assess the relative significance of σ donation and π backdonation, we introduced metrics to evaluate their effective weights, denoted as *W*(σ) and *W*(π), respectively. These metrics are defined as follows:

(1) $$W\left(\sigma \right)=W_{2}+\frac{1}{2}(W_{5}+W_{6})+\frac{1}{3}W_{8}$$

(2) $$W\left(\pi \right)=W_{3}+W_{4}+\frac{1}{2}(W_{5}+W_{6})+W_{7}+\frac{2}{3}W_{8}$$

Table 2 provides a summary of the calculated *W*(σ) and *W*(π) values for all models. These values reaffirm the dominance of σ donation in model I (*W*(σ)>>*W*(π)), while models II and III exhibit an increased relative significance of π backdonation. Notably, in the case of model III, *W*(π) surpasses *W*(σ), which is consistent with our previous finding that π backdonation dominates over σ donation in ferrous heme–CO bonding in P450s [10,11]. Thus, the proximal and equatorial ligands exert substantial influence on amplifying π backdonation. However, it should also be noted that despite the decrease in the *W*_2_ value when transitioning from model II to model III (Table 1), the *W*(σ) value for model III surpasses that of model II (Table 2). This outcome stems from the heightened contribution of the VB structures simultaneously involving both σ and π bonds, specifically Φ_5_ and Φ_6_. Once again, the BOVB calculations yielded qualitatively similar results to the VBSCF calculations, albeit with elevated values for *W*(σ) and *W*(π).

### 2.2. Energy Analysis: Resonance between VB Structures

To gain further insights into the resonance stabilization resulting from the mixing of different VB structures, we conducted energy analyses. Resonance, in essence, involves the mixing of distinct VB structures (diabatic states) that represent different bonding patterns, resulting in a stabilizing effect on the overall wave function. For example, the remarkable stability of benzene finds its rationale in the resonance between the two Kekulé VB structures (alongside other minor ones) [15]. Specifically, we calculated the resonance energy (*RE*) in various states (X) with respect to Φ_1_, the most dominant VB structure, using the following equation:

(3) $$RE\left(X\right)=E\left(\Phi_{1}\right)-E\left(X\right)$$

In this equation, the energy of the variationally optimized $\Phi_{1}$ structure, $E\left(\Phi_{1}\right)$, is calculated as $\langle \Phi_{1}|\hat{H}{|\Phi }_{1}\rangle$ using the Hamiltonian $\hat{H}$. The energy of other states, E(X), is also determined in the same manner. For example, X in this equation is σπ_x_π_y_ in the case of VB(all) calculations, but other states from deactivated VB calculations, such as VB(σ), can also be used as X. It is important to note that a more positive value of *RE* indicates a larger resonance stabilization effect. Table 3a provides a summary of the VBSCF-calculated *RE* values for different states. When we compare the *RE*(σ), *RE*(π_x_), and *RE*(π_y_) values for model I, which gauge the energetic stabilization attributable to respective CT processes, it becomes evident that σ donation plays a notably more substantial role in stabilizing the system compared to π backdonation. However, in model II, the relative significance of σ donation and π backdonation undergoes a notable change. Here, the *RE*(σ) value decreases from 26.98 kcal/mol (model I) to 17.49 kcal/mol (model II), whereas the *RE*(π_x_) and *RE*(π_y_) values increase from 4.03 kcal/mol (model I) to 7.84–7.86 kcal/mol (model II). This trend becomes even more pronounced in the case of model III, which exhibits *RE*(σ), *RE*(π_x_), and *RE*(π_y_) values of 15.77, 12.91, and 12.94 kcal/mol, respectively. Thus, the energetic stabilization for each of these CT routes in model III is of similar magnitude. These results highlight the critical roles played by proximal and equatorial ligands in energetically modulating both the σ-donation and π-backdonation effects, which can be attributed to alterations in the stability of the involved *d*-orbitals within distinct ligand fields [29].

**Figure 3:**
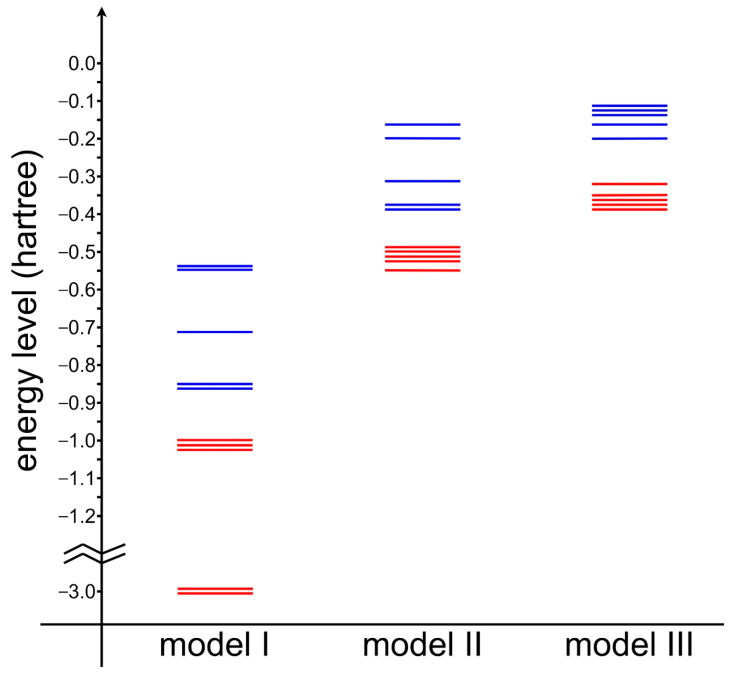
The energy levels of the ten MOs around the frontier orbitals (B3LYP-D3BJ/def2-TZVP(6D,10F)) for models I–III without the CO ligand. The bars for occupied and unoccupied MOs are colored red and blue, respectively.

In Figure 3, we present a comparison of the energy levels of ten molecular orbitals (MOs) in the frontier region (HOMO−4 to LUMO+4) obtained through B3LYP-D3BJ/def2-TZVP(6D,10F) calculations for models I–III after removing their CO ligand (additional details about these MOs can be found in Figure S5). As can be seen from this figure, *d*-orbitals are destabilized to a greater extent when more ligands coordinate to the ferrous center. This results in a higher electron-donating capacity of the iron for π backdonation and a reduced electron-accepting ability for σ donation. Real P450s feature a porphyrin dianion ligand rather than neutral NH_3_ ligands, which would further accentuate the equatorial ligand effect on π backdonation. In fact, our earlier DFT study indicated that in a porphine-based P450 model, π backdonation was approximately twice as significant as σ donation in terms of energetic stabilization [10,11], instead of the approximately 1.6-fold ratio observed in model III.

The data in Table 3 further reveal that the *RE*(σπ_x_), *RE*(σπ_y_), and *RE*(π_x_π_y_) values consistently surpass the corresponding *RE* values for one of their constituent CT routes, i.e., *RE*(σ), *RE*(π_x_), or *RE*(π_y_). In addition, the *RE*(σπ_x_π_y_) value exceeds the values of *RE*(σπ_x_), *RE*(σπ_y_), and *RE*(π_x_π_y_). These findings illustrate the stabilizing influence that results from the mixing of a larger number of VB structures.

*E*(Φ_1_) is identical in our VBSCF and BOVB calculations, and the variational space is broader in BOVB for other states. Hence, the *RE* values calculated using BOVB necessarily exceeded their corresponding VBSCF counterparts, as shown in Table 3b. In essence, this trend signifies that the VB structures involving CT experience greater stabilization in the BOVB framework as a result of the orbital breathing effect. Overall, while the enhancement of the *RE* values in BOVB compared to the VBSCF values is not particularly substantial, relatively significant increases are observed when π_x_ and π_y_ VB structures were simultaneously included in the wave function in model III. It is also noteworthy that both VBSCF and BOVB yield similar enhancements or reductions in *RE* values when transitioning from model I to model III. For instance, in the case of *RE*(π_x_), the enhancement factor is approximately 3.2 in both VBSCF and BOVB results.

To obtain further insights into the resonance stabilization arising from the interaction between different VB structures associated with different CT modes, we conducted supplementary analyses by introducing ∆*RE* values for different combinations of VB structures (Equations (4)–(6)). For instance, ∆*RE*(σπ_x_), as defined in Equation (4), assesses the resonance stabilization arising from the combination of the σ and π_x_ VB structures.

(4) $$\Delta RE\left({\sigma \pi }_{x}\right)=RE\left({\sigma \pi }_{x}\right)-\left[RE\left(\sigma \right)+RE\left(\pi_{x}\right)\right]$$

(5) $$\Delta RE\left({\sigma \pi }_{y}\right)=RE\left({\sigma \pi }_{y}\right)-\left[RE\left(\sigma \right)+RE\left(\pi_{y}\right)\right]$$

(6) $$\Delta RE\left(\pi_{x}\pi_{y}\right)=RE\left(\pi_{x}\pi_{y}\right)-\left[RE\left(\pi_{x}\right)+RE\left(\pi_{y}\right)\right]$$

**Figure 4:**
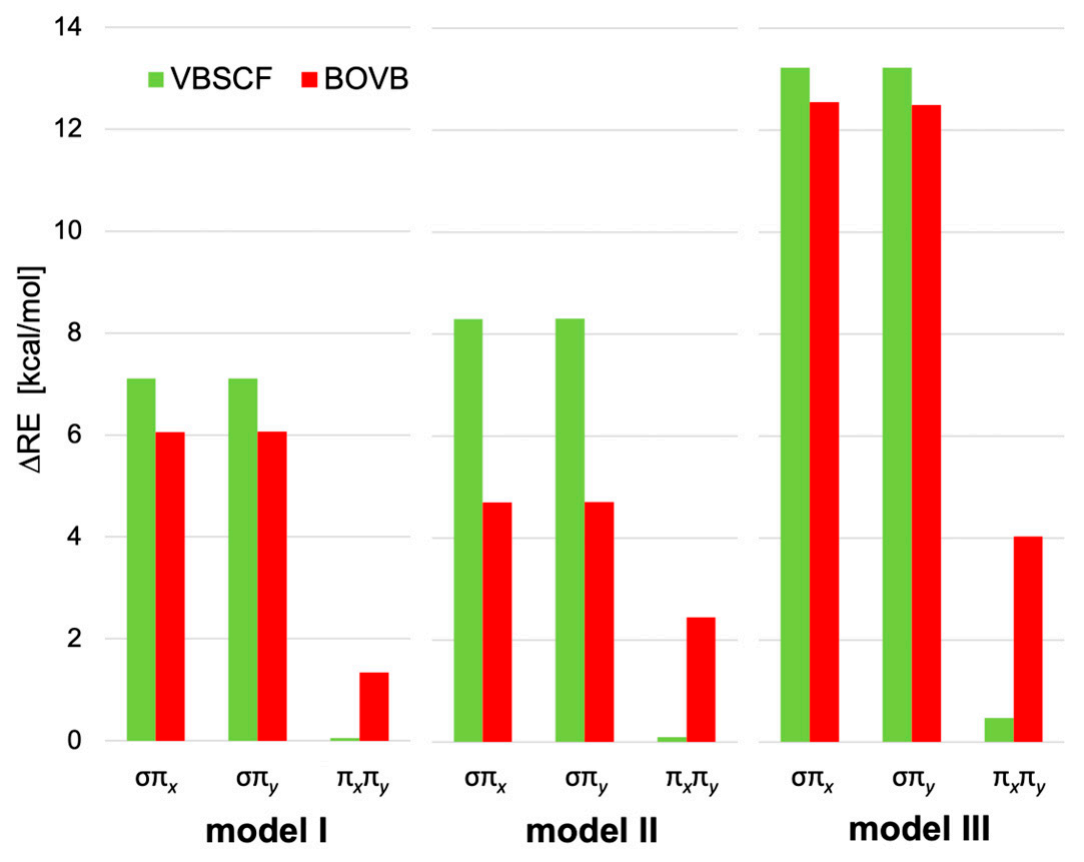
∆*RE* values (in kcal/mol) for models I–III, obtained from VBSCF (green) and BOVB (red) calculations.

A more positive value of ∆*RE* again indicates a greater resonance-stabilizing effect. The calculated ∆*RE* values are presented in Figure 4, revealing a discernible pattern: the σ–π resonance stabilization (∆*RE*(σπ_x_) and ∆*RE*(σπ_y_)) holds notable energetic significance. This observation underscores the existence of a synergistic CT (sCT) effect arising from the interplay between σ donation and π backdonation. The sCT effect does not straightforwardly align with the additive and separate consideration of σ donation and π backdonation, and it cannot be solely explained through ligand-field arguments. Notably, in the case of model III, which most closely mimics a real P450, ∆*RE*(σπ_x_) and ∆*RE*(σπ_y_) display the highest magnitude among all models, indicating that the proximal and equatorial ligands play a prominent role in promoting σ–π resonance. It should also be noted that there are two distinct sets of σ–π resonance interactions (σ–π_x_ and σ–π_y_) due to the presence of two π-backdonation orbital-interaction pairs (Figure 1). Comparatively, many other ligands, unlike CO, can only form a single π-backdonation orbital-interaction pair, resulting in just one set of σ–π resonance. This inherent disparity in sCT leads to a relatively heightened level of energetic stabilization in the ferrous heme–CO complex, distinguishing it from other complexes in terms of its bonding characteristics.

In VBSCF calculations, the ∆*RE*(π_x_π_y_) values exhibit near-zero values across all models. The data indicate that the contribution of π–π resonance to the overall energetic stabilization is notably smaller than that of σ–π resonance. Nevertheless, in BOVB calculations, there is a significant enhancement of π–π resonance (∆*RE*(π_x_π_y_) = 4.03 kcal/mol in model III) compared to the VBSCF value (0.47 kcal/mol). This enhanced π–π resonance contributes to the additional stabilization in ferrous heme–CO bonding. This is especially relevant as many other ligands possess only one π-backdonation orbital-interaction pair and therefore cannot establish any π–π resonance. Consequently, the sCT effect resulting from π–π resonance also contributes to the additional energetic stabilization of ferrous heme–CO bonding. For comparison, we also computed the ∆*RE*(π_x_π_y_) value for acetylene, yielding values of 13.41 kcal/mol (VBSCF) and 13.63 kcal/mol (BOVB) (Table S2). Thus, the energetic significance of π–π resonance is more pronounced in the case of acetylene. This finding also suggests that resonance between different bonding modes may be a widespread phenomenon extending beyond coordination complexes.

**Scheme 3:**
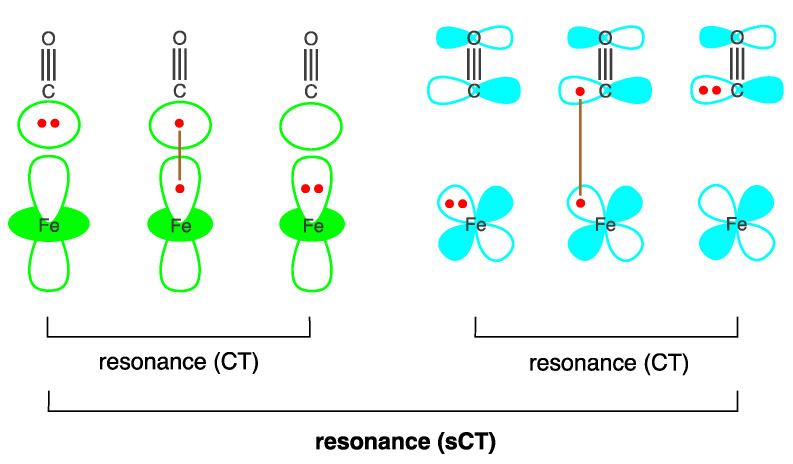
Schematic illustration of the sCT effect, arising from the resonance interaction between different sets of VB structures.

Scheme 3 provides a schematic representation of the sCT effect, a phenomenon uncovered in this study through ab initio VB calculations. sCT involves the contribution of bidirectional CT mechanisms. Firstly, resonance occurs between the VB structures within each of the σ and π frameworks, leading to pure CT stabilization. Secondly, the VB structures from distinct CT frameworks synergistically interact, conferring an additional stabilizing effect. The stabilizing σ–π resonance effect is particularly pronounced in ferrous heme–CO bonding, where two pairs of σ–π resonance can be established. The sCT effect is also feasible between two VB structures associated with π backdonation, although it was observed to possess less energetic significance compared to σ–π resonance.

## 3. Materials and Methods

In our previous study [10], we optimized the geometry of a P450 ferrous heme–CO complex model containing porphine and HS^–^ ligands at the B3LYP-D3BJ/def2-TZVP(6D,10F) level of theory [30,31,32,33,34,35,36]. To better align with the conventional notation of d-orbitals in chemistry such as $d_{z^{2}}$ and $d_{xz}$, we reorientated the optimized geometry such that Fe was placed at the origin, with Fe and the C atom of CO aligned along the *z* axis and one of the equatorial N atoms situated within the *xz* plane. For this purpose, we selected the N atom with the smallest magnitude of the N–Fe–S–H dihedral angle. The resultant model is referred to as model 0. However, applying VB calculations to model 0 was deemed computationally intensive. Therefore, we built three simplified models (I–III) based on model 0, as outlined in Scheme 1. These models retained the essential influence of proximal and equatorial ligands on the iron center while being computationally more manageable. The simplest model, model I, lacked any proximal or equatorial ligands, model II included a proximal HS^–^ ligand, and model III featured both proximal and equatorial ligands (HS^–^ and NH_3_). The atomic positions for models I and II were directly derived from model 0. We followed a similar procedure for constructing model III, but we optimized the positions of the hydrogen atoms within the NH_3_ ligands at the B3LYP-D3BJ/def2-TZVP(6D,10F) level using Gaussian 16 [37], while keeping all other atoms fixed.

Subsequently, we performed ab initio VB calculations for models I–III using the VBSCF method [38,39], employing the 6-31G*(6D,10F) basis set. VBSCF calculations allow for the optimization of both orbitals and VB structure coefficients {*C_K_*} within the total wave function Ψ:

(7) $$\Psi =\underset{K}{\sum }C_{K}\Phi_{K}\;$$

where {Φ*_K_*} are VB structures. For all VB calculations, we utilized the XMVB 3.1 software in both the Xiamen Atomistic Computing Suite (XACS) cloud environment and an installed version [40,41,42]. Additionally, to enhance our understanding of the resonance stabilization in the ferrous heme–CO complex models, we applied VB calculations to acetylene. Orbitals were plotted using Multiwfn and IQmol [43,44].

To effectively analyze the three types of CT effects depicted in Figure 1 employing ab initio VB theory [15,16,17], we classified the AOs from the basis set into different types. This orbital classification enabled us to express VB orbitals in terms of hybrid AOs. Utilizing hybrid AOs, as opposed to delocalized MOs, allowed us to establish a coherent alignment between the resulting electronic configurations and the CT processes across fragments. For the AOs on Fe in model I, four AO groups were defined. The first group encompassed s, $p_{z}$, $d_{x^{2}}$, $d_{y^{2}}$, $d_{z^{2}}$, $f_{z^{3}}$, $f_{x^{2}z}$, and $f_{y^{2}z}$ AOs, which were anticipated to contribute to σ donation. The second group comprised $p_{x}$, $d_{xz}$, $f_{x^{3}}$, $f_{xy^{2}}$, and $f_{xz^{2}}$ AOs, while the third group consisted of $p_{y}$, $d_{yz}$, $f_{y^{3}}$, $f_{x^{2}y}$, and $f_{yz^{2}}$ AOs; these AOs may participate in π backdonation to CO within the *xz* plane (π*_x_*) and the *yz* plane (π*_y_*), respectively. The fourth group was dedicated solely to $d_{xy}$ AOs, which were used to describe a non-bonding electron pair on the ferrous ion.

In our treatment of CO as a single fragment, its AOs were divided into three groups: The first group included all s, $p_{z}$, $d_{x^{2}}$, $d_{y^{2}}$, and $d_{z^{2}}$ AOs from the C and O atoms, which may play active roles in σ donation. The second group encompassed $p_{x}$ and $d_{xz}$ AOs, while the third group comprised $p_{y}$ and $d_{yz}$ AOs, primarily contributing to π backdonation.

A total of six electrons participate in these σ donation and π backdonation processes (two for σ donation and four for π backdonation; Figure 1), and these electrons are collectively referred to as active electrons. VB theory was employed to describe the bonding interaction between Fe(II) and CO, utilizing hybrid AOs that involve six active electrons. It is worth noting that this VB treatment yields numerous VB structures (175 in total) due to different spin-coupling patterns [17,45], including those described in Scheme 2. In Φ_1_, which corresponds to the “dormant state,” the active electrons do not participate in any covalent bonding. In contrast, Φ_2_–Φ_4_ each involve a single HL-type covalent bond, established either in the σ or the π framework, thus incorporating CT across fragments. Φ_5_–Φ_7_ also involve CT but uniquely entail the formation of two HL-type bonding pairs. In Φ_8_, CT transpires across all σ-donation or π-backdonation routes. Electrons that were not treated explicitly by VB were described in a doubly occupied MO fashion, utilizing hybrid AOs. For models II and III, we adhered to essentially the same AO grouping approach, treating Fe and its proximal and equatorial ligands as one fragment. More details of VB orbital specification can be found in the Supplementary Materials (Figure S1). The VB calculation involving all possible VB structures and six active electrons is referred to as VB(all).

To gain deeper insights, we conducted supplementary VBSCF calculations with the deliberate deactivation of specific CT routes. For instance, when we intentionally excluded the explicit VB treatment of the four electrons in the π-type VB structures, the resultant wave function comprised three VB structures for two active electrons within the σ framework (referred to as VB(σ)), as illustrated by the green orbitals in Scheme 3. These VB structures bear resemblance to the three VB structures (one covalent and two ionic) for the H_2_ molecule, which include the left–right electron correlation for the bond [46]. Alternatively, when we prohibited σ donation, we focused on VB structures associated with the orbitals in the π frameworks (i.e., VB(π*_x_*π*_y_*)). In terms of electron correlation, VB(π*_x_*π*_y_*) in VBSCF captures a larger portion of non-dynamical correlation than VB(π*_x_*) or VB(π*_y_*). The initial guess for these calculations was derived from the preceding VB(all) calculation. This deactivation approach allowed us to evaluate the extent of stabilization attained through the resonance mixing of different VB structures for different CT routes. It should be noted that the contribution of the two-electron-transferred state (e.g., the third structure in Scheme 3 in the case of VB(σ)), was typically very small.

To effectively incorporate dynamical electron correlation into our wave functions, we extended our calculations with the BOVB method [46,47,48], building upon the VBSCF results. Again, we concisely described the non-VB electrons using a single set of MOs, focusing our explicit VB treatment on the active electrons. In this approach, VB orbitals are optimized separately for different VB structures. To enhance computational efficiency, our BOVB calculations selectively incorporated VB structures deemed relevant to either σ donation or π backdonation. In the VB(σ), VB(π*_x_*), and VB(π*_y_*) calculations with BOVB, we considered three distinct VB structures within their respective σ or π routes (see Scheme 3). Meanwhile, the VB(σπ*_x_*), VB(σπ*_y_*), and VB(π*_x_*π*_y_*) calculations comprised 9 (=3 × 3) VB structures each, and VB(σπ*_x_*π*_y_*) calculations encompassed a total of 27 (=3 × 3 × 3) VB structures.

## 4. Conclusions

In summary, our investigation into the nature of ferrous heme–CO bonding within cytochrome P450, employing ab initio VB calculations, has yielded valuable quantum mechanical insights. Firstly, our results underscore the dominance of π backdonation in the P450 ferrous heme–CO complex, primarily attributed to the presence of proximal and equatorial ligands. Furthermore, our VB findings illuminate the pivotal role played by σ–π resonance in stabilizing coordination bonding, which gives rise to the emergence of the stabilizing sCT effect. Notably, the presence of two pairs of σ–π resonance in ferrous heme–CO bonding, in contrast to the presence of just one in many other ligands, reinforces the bonding through augmented sCT. Additionally, one pair of π–π resonance also contributes to the bonding. By highlighting sCT as an adhesive force in coordination bonding, this study introduces a fresh perspective on bonding and opens new avenues of inquiry into coordination chemistry—a foundational field with widespread implications across various subdisciplines of chemistry. 

## Figures and Tables

**Table 1:** Weights of Φ_1_–Φ_8_ (in %) for models I–III, obtained by (**a**) VBSCF-VB(all) and (**b**) BOVB-VB(all) calculations ^a^.

**(a) VBSCF**								
Model	*W*_1_	*W*_2_	*W*_3_	*W*_4_	*W*_5_	*W*_6_	*W*_7_	*W*_8_
I	68.39	20.68	1.23	1.23	2.89	2.89	−0.03	0.03
II	69.26	12.31	3.60	3.61	4.05	4.06	−0.08	0.13
III	50.25	8.59	8.52	8.53	9.27	9.27	−0.15	0.75
**(b) BOVB**								
I	60.22	25.81	2.40	2.40	3.81	3.81	−0.03	0.16
II	58.12	18.52	5.83	5.84	5.03	5.05	−0.08	0.43
III	37.22	14.24	12.74	12.76	9.50	9.52	−0.20	1.84

^a^ The total numbers of VB structures in VB(all) calculations with VBSCF and BOVB are 175 and 27, respectively.

**Table 2:** Effective weights (in %) of σ and π characters, determined via the VBSCF method. Values in parentheses are from BOVB calculations.

Model	*W*(σ)	*W*(π)
I	23.6 (29.7)	5.3 (8.7)
II	16.4 (23.7)	11.3 (16.9)
III	18.1 (24.4)	26.7 (36.0)

**Table 3:** *RE* values (in kcal/mol) for various states, obtained from (**a**) VBSCF and (**b**) BOVB calculations.

(a)							
Model	σ	π_x_	π_y_	σπ_x_	σπ_y_	π_x_π_y_	σπ_x_π_y_
I	26.98	4.03	4.03	38.12	38.12	8.11	49.08
II	17.49	7.84	7.86	33.63	33.65	15.79	49.07
III	15.77	12.91	12.94	41.91	41.93	26.32	65.80
**(b)**							
I	28.53	4.85	4.85	39.45	39.45	11.04	51.07
II	18.91	9.36	9.38	34.43	34.46	21.18	51.48
III	17.12	15.52	15.55	45.20	45.17	35.11	72.93

## Data Availability

All data are fully available upon request from the authors.

## Supplementary Materials

The following supporting information can be downloaded at https://www.mdpi.com/article/10.3390/molecules29040873/s1: Figure S1: Input files for VBSCF-VB(all) calculations; Figure S2: VB orbitals in the (a) σ, (b) π_x_, and (c) π_y_ frameworks, obtained through the VBSCF-VB(all) calculation of model I; Figure S3: VB orbitals in the (a) σ, (b) π_x_, and (c) π_y_ frameworks, obtained through the VBSCF-VB(all) calculation of model II; Figure S4: VB orbitals in the (a) σ, (b) π_x_, and (c) π_y_ frameworks, obtained through the VBSCF-VB(all) calculation of C_2_H_2_; Table S1. Total energies (in hartrees) obtained from (a) VBSCF and (b) BOVB calculations; Table S2. ∆*RE* values (in kcal/mol), obtained from (a) VBSCF and (b) BOVB calculations; Figure S5; Frontier orbitals and their energy levels (in hartrees), obtained through B3LYP-D3BJ/def2-TZVP(6D,10F) calculations for models I-III, after removing the CO ligand; Table S3: XYZ coordinates (Å).
