# Supplementary material for: Synergistic Charge Transfer Effect in Ferrous Heme–CO Bonding within Cytochrome P450
Source: Molecules. 2024 Feb 16;29(4):873. doi: 10.3390/molecules29040873 (PMC10891957; doi:10.3390/molecules29040873)
Supplement: Supplementary file 1 [file molecules-29-00873-s001.zip › molecules-2846763-supplementary.pdf]

1. Hybrid AO specification

Figure S1. Input files for VBSCF-VB(all) calculations.

(a) Model I

```
CO-4s.1 ;;; GUS from 4s.0 ;;; ORB-18:ORB-16 ;;;ORB-17 from 4s.0.1
$CTRL
STR=FULL NAO=6 NAE=6 ISCF=5 IPRINT=3
ORBTYP=HAO FRGTYP=SAO
INT=LIBCINT BASIS=6-31g* NCHARGE=2
GUESS=READ
ITMAX=10000
$END
$FRAG
1*4 2*3
; Fe.
SPZDXXDYYDZZFZZZFXXZFYYZ 1
PXDXZFXXXFXYYFXZZ 1
PYDYZFYYYFXXYFYZZ 1
DXY 1
; CO
SPZDXXDYYDZZ 2 3
PXDXZ 2 3
PYDYZ 2 3
$END
$ORB
1*16 1*6
;core 1s of Fe
1
;core 2s of Fe
1
;core 2p6 of Fe
1
2
3
;core 1s of C and O
5
5
;valence 3s2 3p6 3d6 – 2 pi bonds ;;; the active sigma bond's electrons are from C
1
1
2
3
4
;bonds between C and O
5
6
7
;lone pair of O
```

```
5
;;;;;;;;
;AO
;sigma of Fe
1
;sigma of CO
5
;pi-x
2
6
;pi-y
3
7
$END
```

(b) Model II

```
SH-4s.5 ;;; GUS from 4s.4.1 ;;; add FRAG 1 to ORB-18 ;;; adjust coeff of ORB-18, 1~38 (Fe)
to 0.000...
$CTRL
STR=FULL NAO=6 NAE=6 ISCF=5 IPRINT=3
ORBTYP=HAO FRGTYP=SAO
INT=LIBCINT BASIS=6-31g* NCHARGE=1
GUESS=READ
ITMAX=10000
$END
$FRAG
1*4 2*3 2 1 1
; Fe.
SPZDXXDYYDZZFZZZFXXZFYYZ 1
PXDXZFXXXFXYYFXZZ 1
PYDYZFYYYFXXYFYZZ 1
DXY 1
; CO
SPZDXXDYYDZZ 2 3
PXDXZ 2 3
PYDYZ 2 3
; SH
SPZDXXDYYDZZ 4 5
PXDXZ 4
PYDYZ 4
$END
$ORB
2*10 1*2 2*4 1 4 2*3 1*4 2 1 2 1 2 1
;core 1s of Fe
1 8
;core 2s of Fe
1 8
;core 2p6 of Fe
1 8
```

2 9
3 10
;core 1s of S
1 8
;core 2s of S
1 8
;core 2p of S
1 8
2 9
3 10
;core 1s of C and O
5
5 ;12
;valence Fe 3s2 3p6 3d6 – 2 pi bonds ;;; the active sigma bond's electrons are from C
1 8
1 8
2 9
3 10
4
;valence SH- 3s2 3p6
; S-H sigma bond
1 8 9 10 ;18
; S-Fe sigma bond
1 8
; pi of S ; no pi bond with Fe
2 9
3 10
;bonds between C and O
5
6
7
;lone pair of O
5 ;25
;;;;;;;;
;AO
;sigma of Fe
1 8
;sigma of CO
5
;pi-x
2 9
6
;pi-y
3 10
7
$END

I Model III

nh-54s.0 ;;; GUS from nh-4444s.2 ;;; add FRAG 1,11 to ORB-22 ;;; adjust coeff of ORB-22, 1~38 (Fe), 91~174 (NH3) to 0.000...

```
$CTRL
STR=FULL NAO=6 NAE=6 ISCF=5 IPRINT=3
ORBTYP=HAO FRGTYP=SAO
INT=LIBCINT BASIS=6-31g* NCHARGE=1
GUESS=READ
ITMAX=10000
$END
$FRAG
1*4 2*3 2 1 1 16
; Fe
SPZDXXDYYDZZFZZZFXXZFYYZ 1
PXDXZFXXXFXYYFXZZ 1
PYDYZFYYYFXXYFYZZ 1
DXY 1
; CO
SPZDXXDYYDZZ 2 3
PXDXZ 2 3
PYDYZ 2 3
; SH
SPZDXXDYYDZZ 4 5
PXDXZ 4
PYDYZ 4
; porphine
SPZDXXDYYDZZPXDXZPYDYZDXY 6-21
$END
$ORB
3*3 2*2 3*3 2*2 1*2 3*6 2*2 1 5 3 2*2 1*4 3*16 3 1 2 1 2 1
;core 1s of Fe
1 8 11
;core 2s of Fe
1 8 11
;core 2p6 of Fe
1 8 11
2 9
3 10
;core 1s of S
1 8 11
;core 2s of S
1 8 11
;core 2p of S
1 8 11
2 9
3 10
;core 1s of C and O
5
5
;core 1s of 4N of porphine
1 8 11
```

1 8 11
1 8 11
1 8 11
;valence Fe 3s2 3p6 3–6 - 2 pi bonds ;;; the active sigma b'nd's electrons are from C
1 8 11
1 8 11
2 9
3 10
4
;valence SH- 3s2 3p6
; S-H sigma bond
1 8 9 10 11
; S-Fe sigma bond
1 8 11 ;23
; pi of S ; no pi bond with Fe
2 9 ;24
3 10 ;25
;bonds between C and O
5
6
7
;lone pair of O
5 ;29
;valence 2s2 2p6 of 4N
1 8 11
1 8 11
1 8 11
1 8 11
1 8 11
1 8 11
1 8 11
1 8 11
1 8 11
1 8 11
1 8 11
1 8 11
1 8 11
1 8 11
1 8 11
1 8 11
;;;;;;;;
;AO
;sigma of Fe
1 8 11 ;46
;sigma of CO
5 ;47
;pi-x
2 9
6
;and pi-y

```
3 10
7
$END
```

(d) C_2H_2

```
c2h2-4.0.1 ;;; GUS from c2h2-4.0 ;;; 1;3 ;;; 2;4
$CTRL
STR=FULL NAO=6 NAE=6 ISCF=5 IPRINT=3
ORBTYP=HAO FRGTYP=SAO
INT=LIBCINT BASIS=6-31g* NCHARGE=0
GUESS=READ
ITMAX=10000
$END
$FRAG
2*4 2*4
;CH1
SPZDXXDYYDZZFZZZFXXZFYYZ 1 3
PXDXZFXXXFXYYFXZZ 1 3
PYDYZFYYYFXXYFYZZ 1 3
DXY 1 3
;CH2
SPZDXXDYYDZZFZZZFXXZFYYZ 2 4
PXDXZFXXXFXYYFXZZ 2 4
PYDYZFYYYFXXYFYZZ 2 4
DXY 2 4
$END
$ORB
1*2 1*2 1*6
;core 1s2 of C1, C2
1
5
;valence
;CH bond
1
5
;AO
;sigma
1
5
;pi-x
2
6
;pi-y
3
7
$END
```

2. Additional VB results

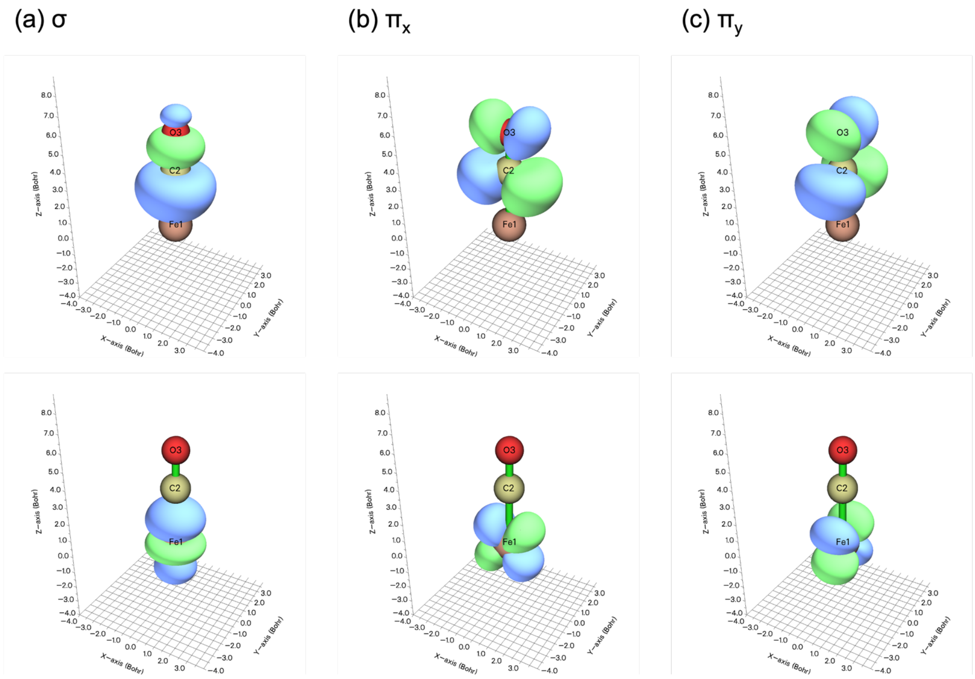

Figure S2. VB orbitals in the (a) σ, (b) π_x, and (c) π_y frameworks, obtained through the VBSCF-VB(all) calculation of model I.

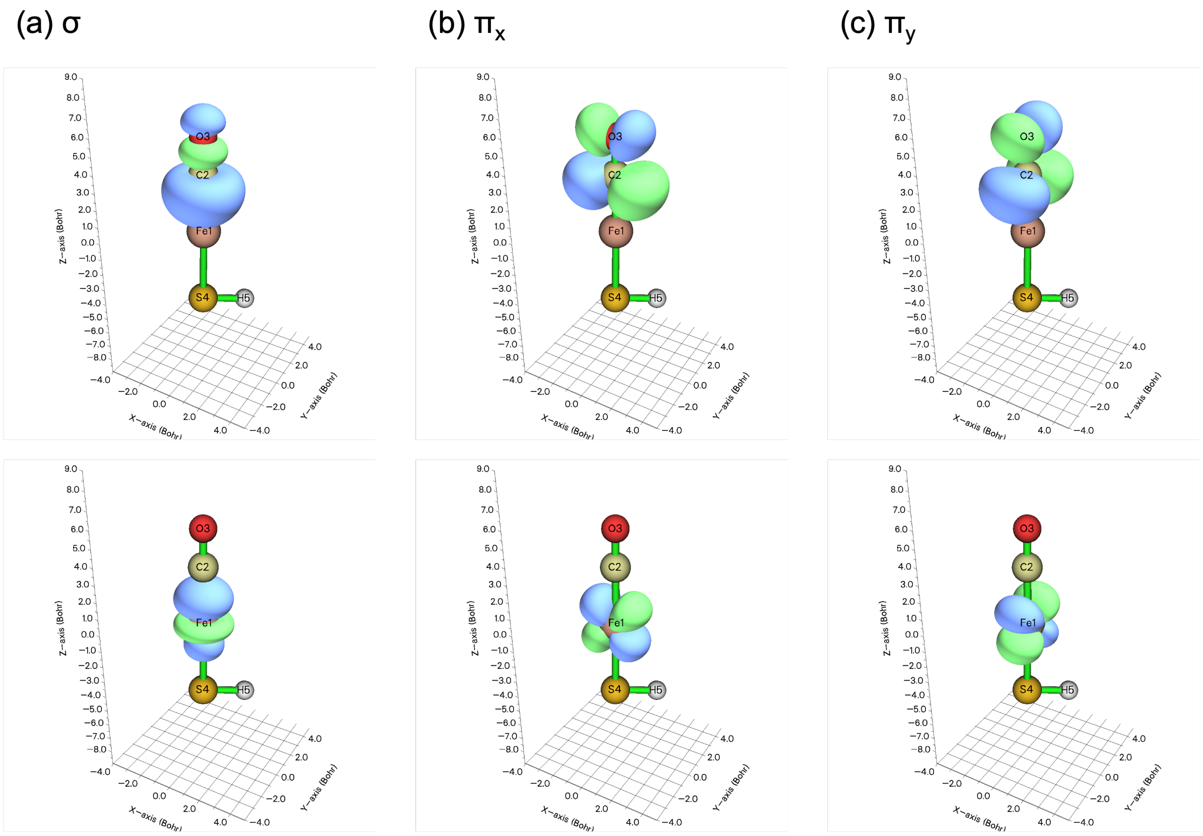

Figure S3. VB orbitals in the (a) σ, (b) π_x, and (c) π_y frameworks, obtained through the VBSCF-VB(all) calculation of model II.

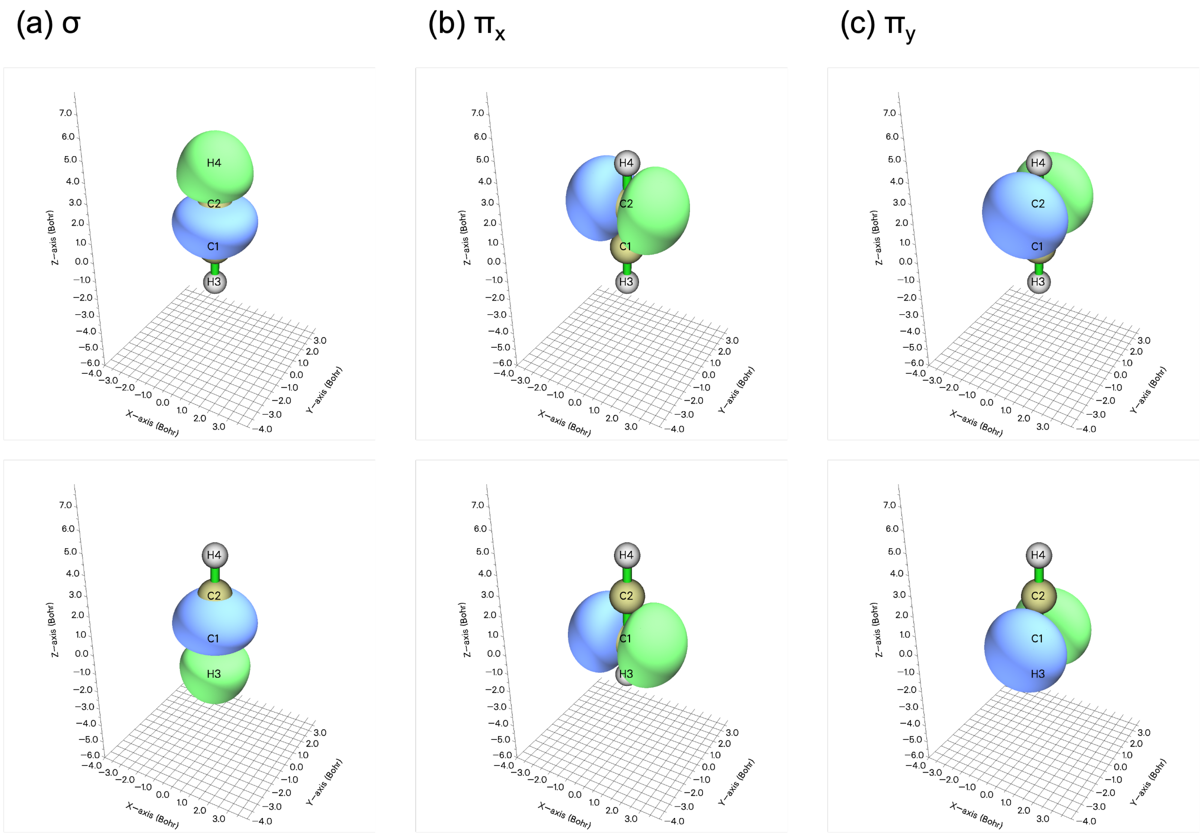

Figure S4. VB orbitals in the (a) σ, (b) π_x, and (c) π_y frameworks, obtained through the VBSCF-VB(all) calculation of C_2H_2.

Table S1. Total energies (in hartrees) obtained from (a) VBSCF and (b) BOVB calculations

(a) VBSCF

model	Φ_1	σ	π_x	π_y
I	-1374.031676	-1374.074675	-1374.038102	-1374.038102
II	-1772.437494	-1772.465361	-1772.449990	-1772.450014
III	-1997.396199	-1997.421329	-1997.416780	-1997.416813
C_2H_2	-76.816834	-76.823409	-76.840155	-76.840155
model	$\sigma\pi_x$	$\sigma\pi_y$	$\pi_x\pi_y$	$\sigma\pi_x\pi_y$
I	-1374.092430	-1374.092430	-1374.044602	-1374.109883
II	-1772.491082	-1772.491117	-1772.462654	-1772.515700
III	-1997.462991	-1997.463021	-1997.438138	-1997.501059
C_2H_2	-76.861107	-76.861107	-76.884840	-76.915894

(b) BOVB

model	Φ_1	σ	π_x	π_y
I	-1374.031676	-1374.077145	-1374.039409	-1374.039407
II	-1772.437494	-1772.467636	-1772.452414	-1772.452440
III	-1997.396199	-1997.423489	-1997.420940	-1997.420974
C_2H_2	-76.816834	-76.823808	-76.840465	-76.840465
model	$\sigma\pi_x$	$\sigma\pi_y$	$\pi_x\pi_y$	$\sigma\pi_x\pi_y$
I	-1374.094543	-1374.094542	-1374.049276	-1374.113064
II	-1772.492363	-1772.492410	-1772.471253	-1772.519539
III	-1997.468229	-1997.468186	-1997.452143	-1997.512422
C_2H_2	-76.866474	-76.866478	-76.885822	-76.932631

Table S2. Δ*RE* values (in kcal/mol), obtained from (a) VBSCF and (b) BOVB calculations

(a) VBSCF

model	Δ*RE*($\sigma\pi_x$)	Δ*RE*($\sigma\pi_y$)	Δ*RE*($\pi_x\pi_y$)
I	7.11	7.11	0.05
II	8.30	8.31	0.09
III	13.23	13.23	0.47
C_2H_2	– [a]	– [a]	13.41

(b) BOVB

model	Δ*RE*($\sigma\pi_x$)	Δ*RE*($\sigma\pi_y$)	Δ*RE*($\pi_x\pi_y$)
I	6.06	6.07	1.34
II	6.15	6.17	2.44
III	12.55	12.50	4.03
C_2H_2	– [a]	– [a]	13.63

[a.] These Δ*RE* values cannot be well defined because the π and σ contributions are effectively incorporated by MOs and thus cannot be clearly turned off in the VB(σ) and VB(π) calculations, respectively. In the VB($\pi_x\pi_y$) calculation of C_2H_2, the MO-described σ-bonding effect can be cancelled.

3. Additional DFT results

(a) Model I

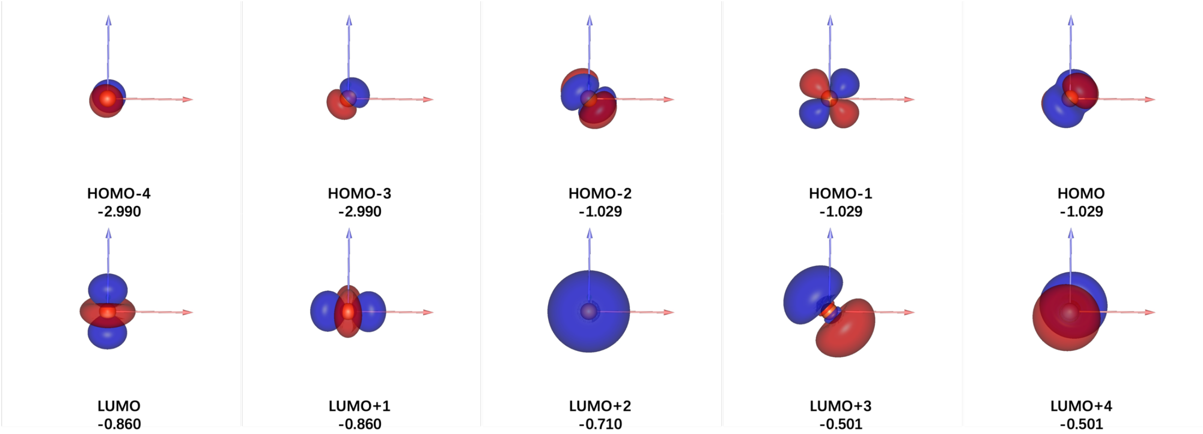

(b) Model II

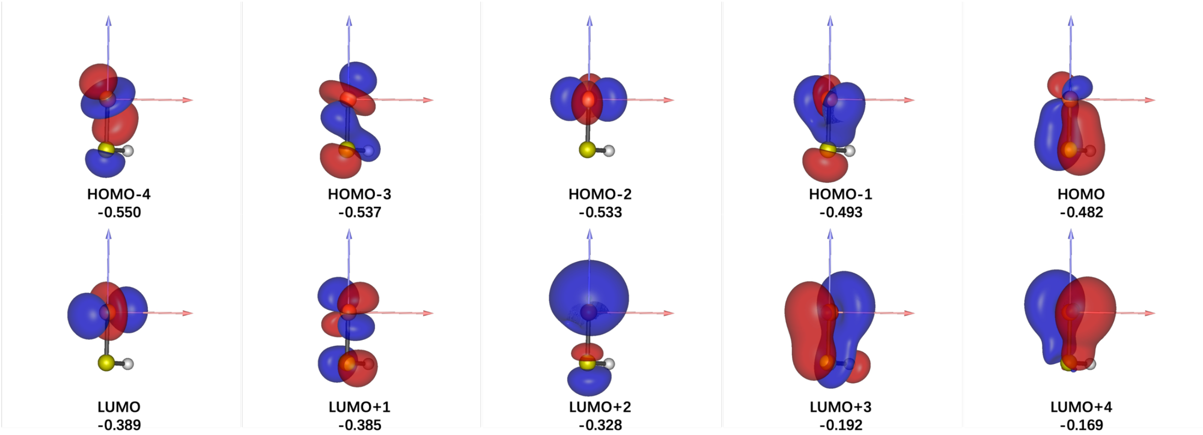

(c) Model III

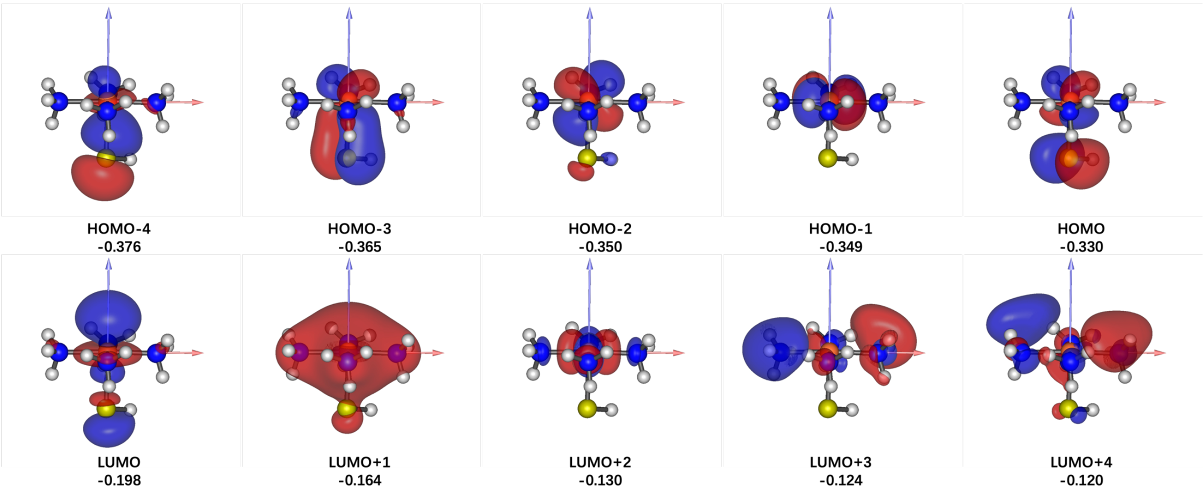

Figure S5. Frontier orbitals and their energy levels (in hartrees), obtained through B3LYP-D3BJ/def2-TZVP(6D,10F) calculations for models I-III, after removing the CO ligand.

4. XYZ coordinates of models

Table S3. XYZ coordinates (Å)

=== Model I ===

Fe	0.000000	0.000000	0.000000
C	0.000000	0.000000	1.796227
O	0.005462	0.005352	2.942542

=== Model II ===

Fe	0.000000	0.000000	0.000000
C	0.000000	0.000000	1.796227
O	0.005462	0.005352	2.942542
S	-0.031421	-0.021799	-2.393336
H	0.962602	0.856852	-2.608789

=== Model III ===

Fe	0.000000	0.000000	0.000000
C	0.000000	0.000000	1.796227
O	0.005462	0.005352	2.942542
S	-0.031421	-0.021799	-2.393336
H	0.962602	0.856852	-2.608789
N	2.022512	0.000000	-0.040263
N	0.005449	2.022900	-0.039572
N	-2.025181	-0.002852	-0.043785
N	-0.007745	-2.025699	-0.040421
H	2.463174	0.908207	0.080583
H	2.291743	-0.321959	-0.969606
H	0.757143	-2.470599	0.460604
H	-0.849346	-2.478202	0.306739
H	-2.477451	0.686319	0.551526
H	-2.471580	-0.886693	0.186905
H	-0.171431	2.286647	-1.008996
H	0.874711	2.473540	0.234242
H	2.480237	-0.609995	0.632669
H	0.091843	-2.292303	-1.020862
H	-2.293680	0.213519	-1.004519
H	-0.712745	2.472062	0.523376

=== C_2H2 ===

C	0.000000	0.000000	0.000000
C	0.000000	0.000000	1.196745
H	0.001436	0.000000	-1.062732
H	0.000480	-0.000472	2.259534
